# Evaluation of vascular events in patients with myeloproliferative syndromes and mutations of either the januskinase-2 or calreticulin gene at the university hospital Krems from 2008 to 2015

**DOI:** 10.18632/oncotarget.23879

**Published:** 2018-01-03

**Authors:** Sarah Hintermair, Elisabeth Zwickl-Traxler, Martin Pecherstorfer, Josef Singer

**Affiliations:** ^1^ Department of Internal Medicine II, University Hospital Krems, Karl Landsteiner University of Health Sciences, Krems an der Donau, Austria

**Keywords:** myeloproliferative syndromes, polycythemia vera, essential thrombocytosis, myelofibrosis, vascular events

## Abstract

Myeloproliferative neoplasms (MPN), classified as polycythemia vera (PV), essential thrombocytosis (ET) and myelofibrosis (MF) are stem-cell derived disorders. Mutations in either the januskinase-2 (JAK-2) or the calreticulin (CALR) gene are characteristic for MPN and may result in enhanced proliferation of red blood cells, white blood cells and platelets, and thus increase the risk for vascular events.

This study is a retrospective and descriptive analysis of records of patients, who underwent treatment for myeloproliferative syndromes at the Department of Hemato-Oncology of the University hospital Krems from 2008 to the end of 2015. Out of 250 patients, who were suspected for MPN, 51 patients displayed a JAK-2 V617F mutation. These were analyzed with regard to their blood values, gender, age at diagnosis, therapy and vascular events before and after diagnosis (during therapy). Of the 51 patients diagnosed with MPN and a JAK-2 V617F mutation, 33 suffered from PV, 15 from ET and 3 from MF. More men than women were diagnosed with MPN and the median age at diagnosis was 72 years. Acetylsalicylic acid, phlebotomy and Hydroxyurea were the most frequent therapies applied. In our study cohort, the most common vascular events were acute coronary syndrome and transitory ischemic attack. Thromboembolic events were effectively reduced by MPN therapy while no elevation in bleeding events could be observed.

## INTRODUCTION

### Myeloproliferative neoplasms (MPN)

Myeloproliferative neoplasms (MPN) are characterized as clonal hematopoietic stem cell diseases with increased proliferation of one or more cell lines of the myeloid lineages [1]. BCR-ABL-negative MPNs can be differentiated in polycythemia vera (PV), essential thrombocytosis (ET) and myelofibrosis (MF) [1–2]. The incidence of MPN is about 6-10/100.000 [1]. Morphologically, MPN are defined by enhanced cellularity of the bone marrow with increased hematopoiesis of red blood cells (RBC), white blood cells (WBC) or platelets (PLT) [3]. Recently, two gene mutations leading to myeloproliferative disorders have been identified: mutations in the Januskinase-2 (JAK-2) and the calreticulin (CALR) gene [1–4]. The JAK-2 V617F mutation is a hallmark for diagnosis of PV, ET and MF. It triggers auto-phosphorylation of this protein without binding of signaling molecules like erythropoietin (EPO), thrombopoietin (TPO) or granulocyte colony-stimulating factor (G-CSF) and thus leads to enhanced proliferation of blood cells [2–5]. This results in a higher risk for vascular events like thrombosis, myocardial infarction, stroke or GI bleeding [4]. Brodmann et al. describe that the number of vascular events, defined as the number of vascular complications per 100 patient years, is 16.7 for patients with PV, 13.8 for MF and 7.5 for ET and thereby significantly above the average of healthy people of the same age [6].

In JAK-2 negative patients, mutations of the calreticulin (CALR) gene are common [7] and also lead to increased production of blood cells [8–10]. The pathophysiological mechanisms of PV, ET and MF include myeloproliferation of hematologic stem cells with changes in bone marrow and the spleen [2]. Besides JAK-2 and CALR mutations, also mutations in the myeloproliferative leukemia (MPL) gene, which is encoding the thrombopoietin receptor, were described in MPN patients [11]. JAK-2 V617F activates signaling through the EPO-receptor, the TPO-receptor as well as the G-CSF receptor and can thus be associated with erythrocytosis, thrombocytosis, and neutrophilia. CALR mutations mainly activate the TPO-receptor and at lower levels the G-CSF-receptor, leading to phenotypes with elevated PLT and WBC [5]. Consequently, JAK-2 V617F can be found in 95% of PV patients, and 50–60% of ET and MF patients, whereas CALR mutations can be identified in 20–25% of ET and 25–30% of MF patients [5].

The classification of patients into one of the three disease subtypes is important as clinical outcomes and life expectancy differ greatly between the groups [1].

Different therapy options exist to reduce the rate of vascular events: phlebotomy, thrombocyte aggregation inhibitors, cytoreductive therapy with Hydroxyurea, Anagrelide or Interferon-α, JAK-2 inhibition and allogeneic stem cell transplantation [12–13].

### Polycythemia vera (PV)

PV is (as described previously) a chronic myeloproliferative neoplasm with enhanced proliferation of blood cells independent of signaling molecules [4]. PV is also characterized by erythrocytosis, thrombocytosis, leukocytosis, splenomegaly, bleeding, microcirculatory symptoms, pruritus and risk of leukemic or fibrotic transformation [14]. Increased hematocrit and hemoglobin values are characteristic for PV [12]. Due to the increased hyperviscosity of the blood, the risk for thrombosis is elevated [4]. The annual incidence of PV raises with higher age and is about 0.4-2.8/100.000 per year [15]. The median age at diagnosis is around 70 years [12, 16] with a male predominance [17].

Diagnosis of PV requires the combination of clinical investigation, laboratory testing and bone marrow specimens, according to the 2016 WHO criteria [14]. These criteria include hemoglobin and hematocrit threshold values for the diagnosis of PV of 16.5g/dL and 49% for men and 16g/dL and 48% for women, respectively [14]. Enlargement of the spleen is also an important diagnostic criterion [4, 18]. Moreover, determination of erythropoietin (EPO) levels can be helpful for differential diagnosis, as patients with lung diseases and hypoxemia can also develop secondary polyglobulia [19]. Here, the European Society for Medical Oncology (ESMO) Guidelines regard sub-normal sEPO levels in patients with elevated RBC as minor diagnostic criterion [20].

Nearly all patients suffering from PV are diagnosed with a mutation of the Januskinase-2 gene. Of these, 98% display the JAK-2 V617F mutation; the remaining 2% comprise of other JAK-2 mutations including exon 12 mutations [12, 14, 21]. JAK-2 exon 12-mutated PV is more common in younger patients [12]. In patients with a negative mutation analysis for JAK-2, mutations in the calreticulin gene (CALR) have been reported [3, 9–10, 18]. The typical morphological features in bone marrow specimens include trilineage hyperplasia with left shift and pleomorphic megakaryocytic hyperplasia with atypical features [12, 14].

The most common symptoms of PV are related to hypertension and vascular problems caused by the increased blood cell mass [18]. These symptoms include plethora, pruritus, splenomegaly, weight loss, weakness and sweating [4]. Furthermore, gout, hepatomegaly and chest pain can occur as well [4, 12]. Venous and arterial vascular events, like stroke, myocardial infarction or thrombosis are common and can be the first signs of expression of PV [18]. In general, spleen and liver are the primarily affected organs, but any organ can be damaged due to the high blood cell values and consecutive circulatory disturbances [18, 22–23].

The major goal in PV treatment is to prevent vascular events, without raising the risk of bleeding. Thus, the type of treatment has to be chosen individually based on the patient’s risk for vascular events [3, 14]. Patients suffering from PV can be distributed to two risk groups: high-risk, including patients older than 60 years or patients with a vascular event in their history, and low-risk (younger than 60 years and no vascular event prior to diagnosis) [14].

For low-risk patients, low-dose Acetylsalicylic acid (ASA; range 40 mg/d-100 mg/d) has beneficial effects in preventing venous and arterial thrombosis [14, 24]. It could also be shown that low-dose ASA is favorable with regard to microvascular disturbances like headache, pruritus and tinnitus [14, 22, 25]. Patients who are resistant to low-dose ASA or who are at a higher risk for thrombosis should take ASA twice-daily instead of once daily [14, 26]. Alternatively, other anti-platelet drugs can be used in patients with resistance to ASA [14]. Furthermore, phlebotomies should be performed in both risk groups in order to maintain hematocrit values below 45% [3, 14]. Cytoreductive drugs are not recommended for low-risk patients [14].

As mentioned before, high-risk patients are defined as older than 60 years and/or the presence of any prior arterial or venous vascular event [14, 27]. The recommended treatment algorithm for patients of the high-risk category comprises low-dose ASA, phlebotomy (again in order to maintain hematocrit values below 45%) and Hydroxyurea [14, 27]. Hydroxyurea further decreases the risk of vascular events by keeping the PLT count in a normal range, too [14]. It is suitable to use ASA twice a day in high-risk patients suffering from arterial vascular events [14, 26]. In contrast, for high-risk patients suffering from venous vascular events, systemic anticoagulation and once daily low-dose ASA is advised [14]. Patients showing severe side effects or intolerant to Hydroxyurea should be treated with Interferon-α [28], Busulfan [14] or a JAK-2 inhibitor [24].

### Essential thrombocytosis (ET)

Essential thrombocytosis (ET) is a subgroup of MPN, which is characterized by an increased number of platelets, enhanced megakaryocyte count in bone marrow and higher risk for vascular events such as thrombosis or bleeding events [1, 29]. The incidence of ET is approximately 2.5/100.000 per year [30]. Most patients with ET are diagnosed at an age of around 65 years [16] and slightly more women than men are suffering from ET [12, 16–17].

Since there is no known genetic or biological marker specific for ET, other diseases linked with high PLT counts, such as PV, MF, infectious diseases, hemorrhage or other neoplasms must be excluded prior to the diagnosis of ET [3, 14, 30]. Only 60-65% of all patients with ET harbour a JAK-2 mutation [1, 31], but the World Health Organization (WHO) describes a mutation of either JAK-2 or the CALR gene as an important diagnostic hallmark for ET [1, 14]. The diagnosis of ET is confirmed when PLT counts are above 400 G/L, the bone marrow specimen reveals atypical megakaryocytes and there is no other myeloid neoplasm [1, 30].

At the time of diagnosis, most patients are asymptomatic; some are diagnosed because of vascular events or hemorrhages. Due to an increased PLT count, microvascular events like ischemic attacks and gangrene can occur, while thrombosis of the big blood vessels can cause splenic or hepatic vein thrombosis [22–23]. ET can be free of symptoms for a long period of time, but it is a risk factor for vascular events [2–3, 18, 30].

Again, the major treatment goal in ET is to reduce the risk of vascular events [14, 31]. Patients suffering from ET are administered into three risk-categories: very low risk (patients younger than 60 years, no vascular event before, no JAK-2 mutation), low risk (patients younger than 60 years, no vascular event before, JAK-2 mutation present) and high risk (vascular event before or age older than 60 years with confirmed JAK-2 mutation) [14]. The treatment algorithm for patients with ET is similar to the algorithm of PV. Patients in the very low-risk group can be just observed, while patients in the low-risk group are treated with low-dose ASA once or twice daily [14, 32]. Patients in the high-risk category should receive Hydroxyurea and low-dose ASA as a first line treatment [14, 33].

### Myelofibrosis (MF)

Myelofibrosis (MF) is the third subgroup of MPN. It is characterized by enhanced proliferation of megakaryocytes and granulocytes in the bone marrow with variable degrees of cytopenias [1, 34], displacement of fibrous connective tissue and extramedullary hematopoiesis in the end stages of MF [1, 35]. The incidence of MF is about 0.6/100.000 person-years [36]; the median age at diagnosis is 70 years [37] and both genders suffer equally from MF [35].

In 60–65% of MF patients, JAK-2 mutations can be detected, which is also a major diagnostic criterion according to the WHO [1, 14]. There can be a progression from a prefibrotic phase (preMF), which shows histologically similarities to ET by hypercellular bone marrow with megakaryocytic proliferation. Amongst other discrimination criteria, in ET megakaryocytes are enlarged and mature, whereas megakaryocytes in preMF usually display atypia and are accompanied by reticulin or collagen fibrosis [38].

Many organs can function as sites of extramedullary hematopoiesis in the fibrotic phase like the spleen, liver, lymph nodes, kidneys, dura mater, GI tract, lung and pleura [39]. Symptoms of MF include fatigue, weakness, splenomegaly weight loss, bleeding, night sweats, fever and bone pain [40].

The major cause of death is bone marrow failure and therefore, allogeneic stem cell transplantation is the only curative treatment option for MF [3].

### Aims

As patients with MPN have an increased risk, prevention of vascular events is the main therapy goal in patients with MPN. Therefore, our study determined and analyzed vascular events (as listed in Table 1) in our MPN patient cohort before diagnosis and during therapy.

**Table 1:** List of investigated vascular events

Arterial events	Venous events	Bleeding events
Acute coronary syndrome	Deep vein thrombosis	Haemorrhagic insult
Transitory ischemic attack	Pulmonary embolism	Gastrointestinal bleeding
Embolic insult	Mesenteric vein thrombosis	

## RESULTS

**Figure 1:**
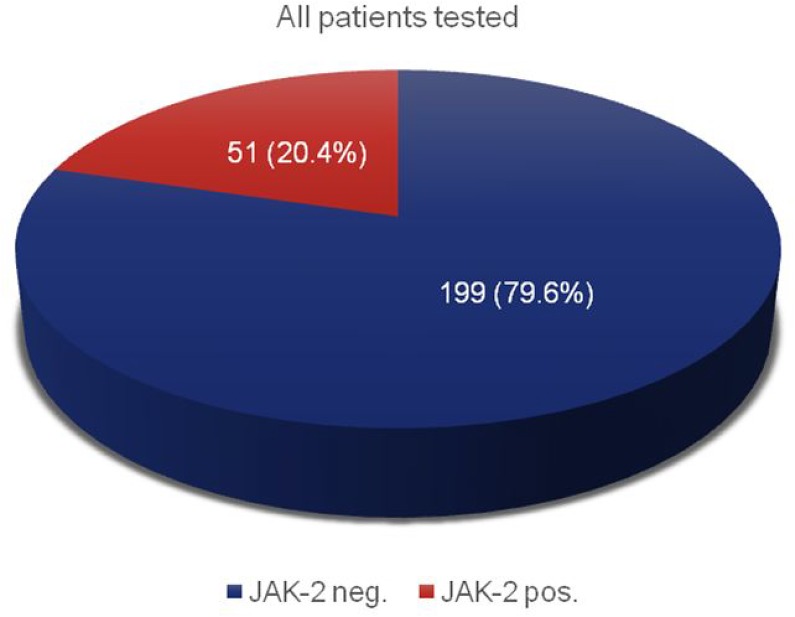
JAK-2 mutation analysis of all suspected MPN patients; *n* = 250

Table 2 gives an overview of all patients, which were examined for myeloproliferative syndromes. The total number of electronic health records was 250 with a gender distribution of 152 (60.8%) men and 98 (39.2%) women. All patients were tested for mutations of the JAK-2 gene. 14 patients were additionally tested for mutations in the CALR gene. 51 (20.4%) patients displayed a mutation of JAK-2 V617F (Figure 1). By contrast, no patient with CALR mutations was identified.

**Table 2:** Overview of all patients tested

	All patients tested
Patients; *n* (%)	250 (100)
Male sex; *n* (%)	152 (60.8)
Female sex; *n* (%)	98 (39.2)
JAK-2 pos. (%)	51 (20.4)
JAK-2 neg. (%)	199 (79.6)
CALR pos.	0

**Figure 2:**
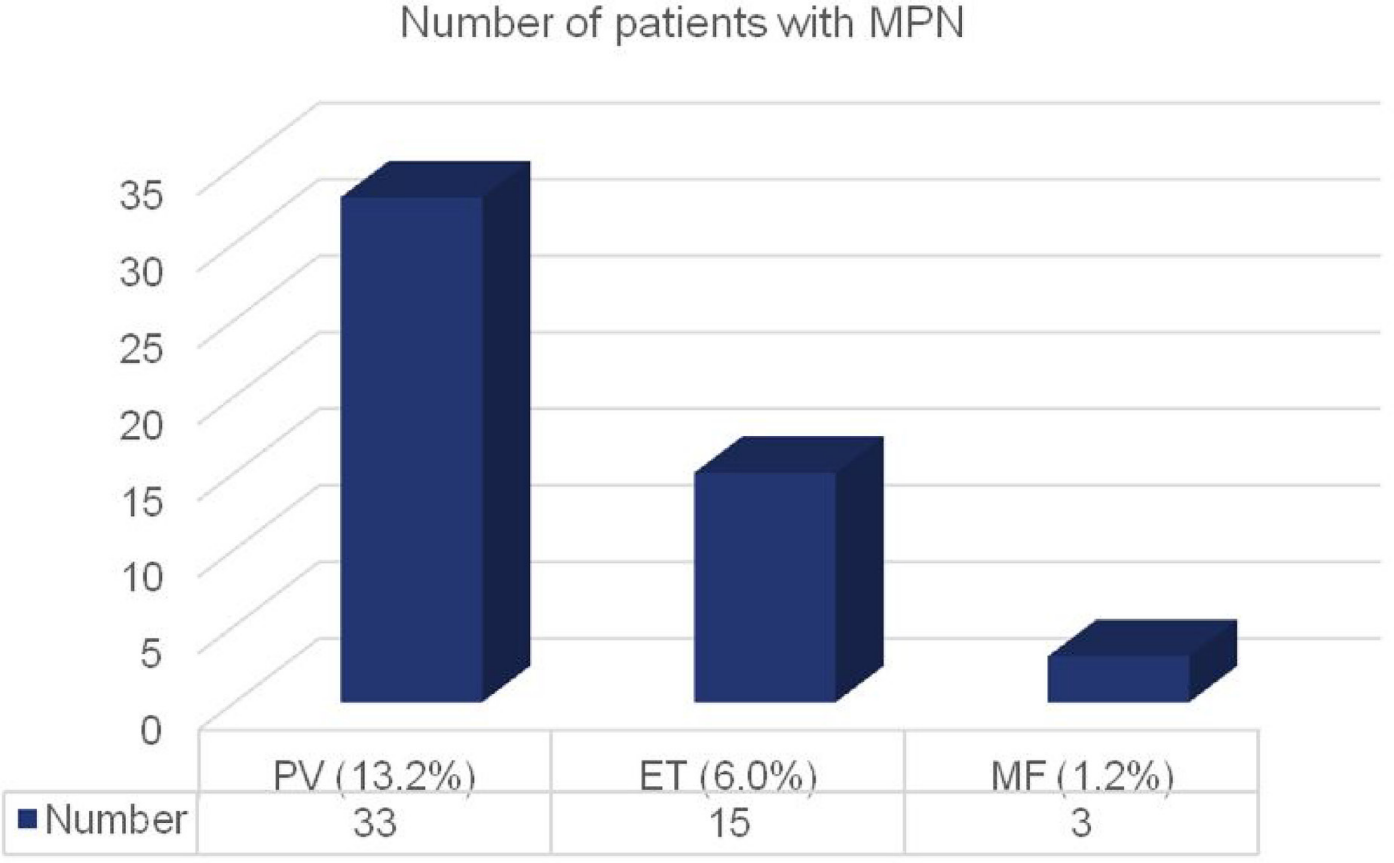
Number of patients with MPN distributed to the three disease subtypes

General characteristics of all patients with MPN are shown in Table 3. As shown in (Figure 2), 33 patients (13.2% of all tested) suffered from PV, 15 (6.0%) patients were diagnosed with ET and 3 (1.2%) patients were diagnosed with MF.

**Table 3:** General characteristics of all patients with MPN at diagnosis

	Patients with MPN	PV	ET	MF	Normal range
Patients; n total (% of all patients tested)	51 (20.4)	33 (13.2)	15 (6.0)	3 (1.2)	
Male sex; n (% of all patients tested)	36 (14.4)	24 (9.6)	10 (4.0)	2 (0.8)	
Female sex; n (% of all patients tested)	15 (6.0)	9 (3.6)	5 (2.0)	1 (0.4)	
Age at diagnosis (years); median (min-max)	72.0 (40–88)	71 (40–88)	74 (53–84)	73 (72–82)	
Hematocrit (%); median (min-max)	46.25 (27.9–64.0) *n =*46	52.5 (31.8–64.0) *n =*30	39.9 (29.7–45.2) *n =*13	32.0(27.9–41.4) *n =*3	35–45.5
RBC (T/l); median (min-max)	5.3 (3.3–8.0) *n =*46	6.2 (3.7–8.0) *n =*29	4.4 (3.7–4.9) *n =*14	3.4 (3.3–4.2) *n =*3	3.85–5.2
Platelets (G/l); median (min-max)	513.0 (99.0–1441.0) *n =*47	453.0 (150.0–1001.0) *n =*30	808.5 (506.0–1441.0) *n =*14	161.0 (99.0–360.0) *n =*3	160–370
WBC (G/l); median (min-max)	11.7 (5.5–30.8) *n =*47	13.5 (7.8–30.8) *n =*30	11.1 (7.5–22.5) *n =*14	6.5 (5.5–7.3) *n =*3	3.6–10.5

**Figure 3:**
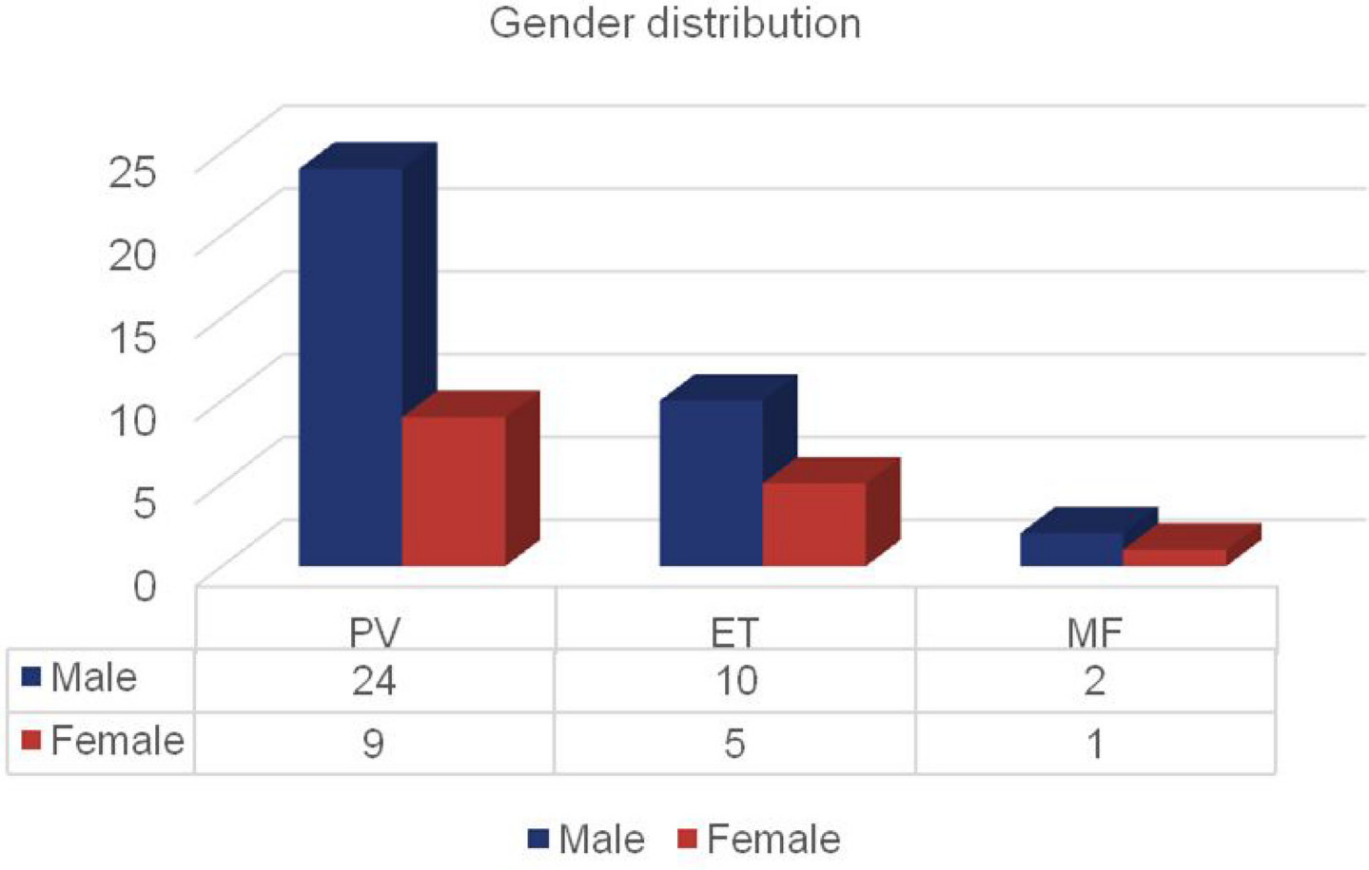
Number of patients with MPN distributed by disease subgroup and gender Bars represent the numbers of patients distributed to PV, ET and MF divided by gender.

In our MPN patient cohort, gender distribution was shifted towards male sex. In the subgroup of PV 24 men and 9 women were diagnosed. 10 men and 5 women were identified for ET, while 2 male and 1 female patients were suffering from MF (Figure 3).

**Figure 4:**
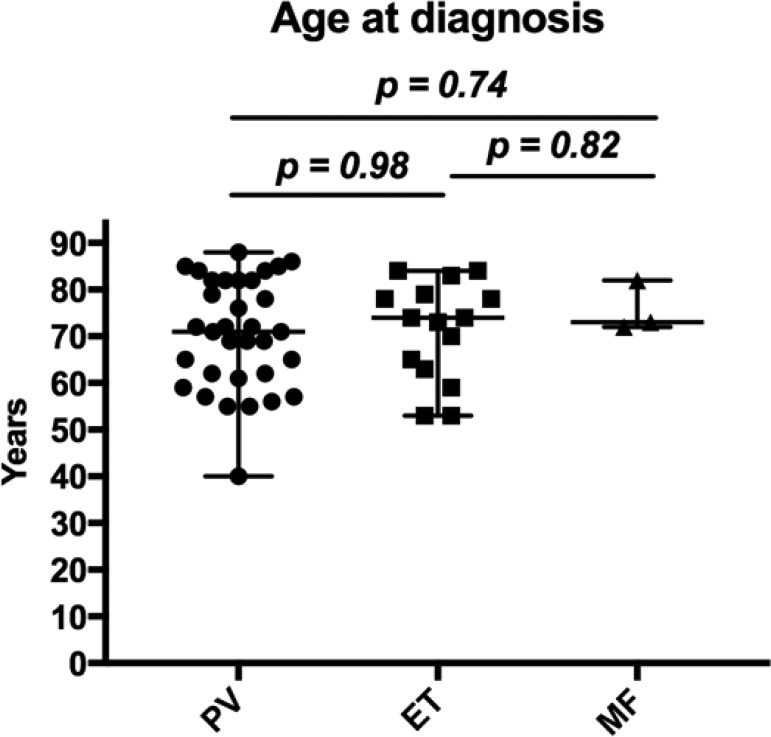
Age at diagnosis of patients with MPN Whiskers show the range from minimum to maximum age.

The median age at diagnosis was 72; in detail 71 years for patients with PV, 74 for patients with ET and 73 for patients with MF (Figure 4).

Table 2 also shows the difference in laboratory values among patients with MPN.

**Figure 5:**
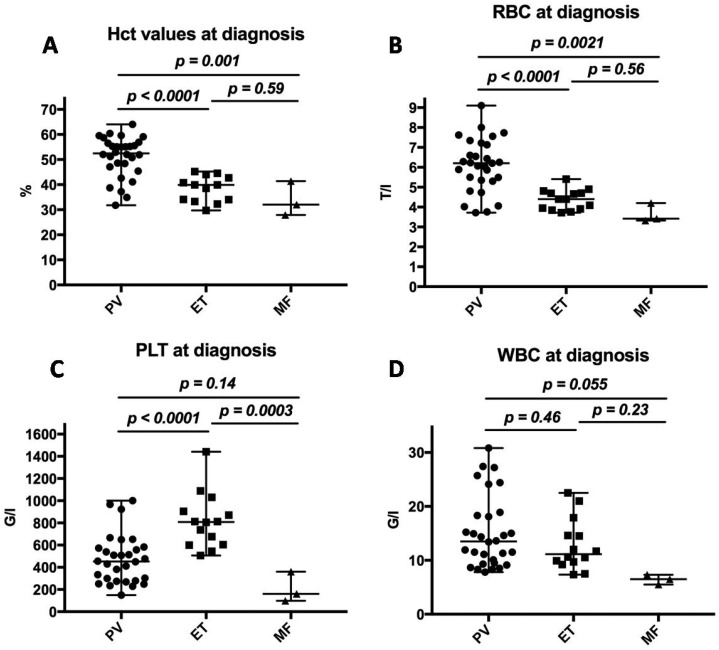
Laboratory results of patients diagnosed with MPN Values are displayed in percent; whiskers represent the range from minimum to maximum values. (**A**) Hematocrit values in patients with MPN at diagnosis. (**B**) Red blood cell counts in patients with MPN at diagnosis. (**C**) Platelet counts in patients with MPN at diagnosis. (**D**) White blood cell counts in patients with MPN at diagnosis.

Patients diagnosed with PV presented at first diagnosis with median Hct values of 52.5%, patients with ET with 39.9% and MF with 32.0% (Figure 5A). The median number of RBC was elevated in patients with PV (6.2 T/l), within in the normal range in patients with ET (4.4 T/l) and decreased in patients with MF (3.4 T/l; Figure 5B). The different values of PLT (G/l) in patients with MPN are shown in Figure 5C; here, patients with PV had median PLT values of 453.0, patients with ET 808.5 and with MF 161.0. For WBC (G/l) PV patients displayed median values of 13.5, ET patients 11.1 and MF patients 6.5 (Figure 5D).

The different treatment options applied in our patient cohort are presented in Table 4. 24 (72.7%) of all patients with PV were treated with phlebotomies, 28 (84.8%) patients took ASA as a daily therapy and 25 (75.8%) were treated with Hydroxyurea. 5 (15.2%) patients suffering from PV received Interferon-alpha and no one underwent stem cell transplantation. Furthermore, 6 (18.2%) people were treated with Anagrelide and 1 patient with Ruxolitinib.

**Table 4:** Frequency of different treatments in patients with MPN

	PV	ET	MF
Phlebotomy; *n* (%)	24 (72.7)	0	0
Acetylsalicylic acid, *n* (%)	28 (84.8)	11 (73.3)	1 (33.3)
Hydroxyurea; *n* (%)	25 (75.8)	10 (66.7)	0
Ruxolitinib; *n* (%)	1 (3.0)	0	0
Interferon alpha; *n* (%)	5 (15.2)	2 (13.3)	0
Anagrelide; *n* (%)	6 (18.2)	7 (46.7)	0
Stem cell transplantation; *n* (%)	0	0	0

In the subgroup of ET, ASA was frequently applied, too. 11 (73.3%) patients received ASA, whereas 10 (66.7%) patients got Hydroxyurea. Anagrelide was administered to 7 (46.7%) patients with ET.

One patient (33.3%) suffering from myelofibrosis (*n* = 3) received Acetylsalicylic acid as treatment.

As depicted in Table 5, differences in the rate of vascular events before and after diagnosis were observed in patients with MPN: before the diagnosis of PV, 7 (21.2%) patients suffered from an acute coronary syndrome (ACS), whereas after diagnosis/during therapy these events could be reduced to 3 (9.1%). A transitory ischemic attack (TIA) occurred in 2 (6.1%) patients before and in one patient (3.0%) after the diagnosis. Other events that could be seen in one patient were: deep vein thrombosis (DVT) and pulmonary embolism (PE) before and DVT, PE, mesenteric vein thrombosis (MVT) and embolic insult after the diagnosis of PV. Moreover, two patients (6.1%) endured a hemorrhagic insult before diagnosis, an event that was not seen under specific therapy. Additionally, two patients presented with gastrointestinal (GI) bleeding before and after diagnosis of PV.

**Table 5:** Number of vascular events before and after diagnosis in patients with MPN

	PV		ET		MF	
	Before	After	Before	After	Before	After
Acute coronary syndrome; *n* (%)	7 (21.2)	3 (9.1)	1 (6.7)	0	0	0
Transitory ischemic attack; *n* (%)	2 (6.1)	1 (3.0)	1 (6.7)	0	0	0
Embolic insult; *n* (%)	0	1 (3.0)	0	0	0	0
Deep vein thrombosis; *n* (%)	1 (3.0)	1 (3.0)	0	0	0	0
Pulmonary embolism; *n* (%)	1 (3.0)	1 (3.0)	1 (6.7)	1 (6.7)	0	0
Mesenteric vein thrombosis; *n* (%)	0	1 (3.0)	2 (13.3)	0	0	0
Hemorrhagic insult; *n* (%)	2 (6.1)	0	1 (6.7)	0	0	0
Gastrointestinal bleeding; *n* (%)	2 (6.1)	2 (6.1)	0	0	0	0

In the ET subgroup one patient (6.7%) suffered from an ACS, one from a TIA, one from a PE and one experienced a hemorrhagic insult before diagnosis. Under treatment only one further event occurred: one patient developed a second PE. The most frequent vascular event in our patients with ET, however, was mesenteric vein thrombosis prior to diagnosis (*n* = 2, 13.3%).

None of our ET patients experienced an embolic insult, DVT or GI bleeding neither before nor after diagnosis of ET. No vascular event was reported in the small subgroup of MF patients.

Table 6 gives an overview on the correlation between the type of medication and specific vascular events during therapy in patients with MPN. The biggest reduction in vascular events could be seen in the groups treated with phlebotomies, ASA and Hydroxyurea. However, in these groups the rate of GI bleeding was higher than before treatment.

**Table 6:** Vascular events before and after diagnosis in patients with MPN grouped by treatment

Medication	ACS		TIA		Embolic insult		DVT		PE		MVT		Hemorrhagic insult		GI bleeding	
	Before	After	Before	After	Before	After	Before	After	Before	After	Before	After	Before	After	Before	After
Phlebotomy; *n* (%) *n =*24	6 (25.0)	3 (12.5)	1 (4.2)	0	0	1 (4.2)	1 (4.2)	1 (4.2)	0	1 (4.2)	0	1 (4.2)	1 (4.2)	0	1 (4.2)	2 (8.3)
Acetylsalicylic acid; *n* (%) *n =*40	7 (17.5)	3 (7.5)	3 (7.5)	1 (2.5)	0	1 (2.5)	1 (2.5)	1 (2.5)	1 (2.5)	1 (2.5)	1 (2.5)	1 (2.5)	1 (2.5)	0	0	2 (5.0)
Hydroxyurea; *n* (%) *n =*35	5 (14.3)	3 (8.6)	3 (8.6)	0	0	1 (2.9)	1 (2.9)	1 (2.9)	1 (2.9)	1 (2.9)	1 (2.9)	1 (2.9)	2 (5.8)	0	1 (2.9)	2 (5.8)
Ruxolitinib; *n* (%) *n =*1	0	1 (100)	0	0	0	1 (100)	0	1 (100)	0	0	0	0	0	0	0	1 (100)
Interferon alpha; *n* (%) *n =*7	0	0	1 (14.3)	0	0	0	1 (14.3)	0	0	1 (14.3)	0	1 (14.3)	0	0	1 (14.3)	0
Anagrelide; *n* (%) *n =*13	1 (7.7)	1 (7.7)	1 (7.7)	1 (7.7)	0	1 (7.7)	0	1 (7.7)	0	1 (7.7)	0	1 (7.7)	0	0	0	1 (7.7)
Stem cell transplantation; *n* (%) *n =*0	0	0	0	0	0	0	0	0	0	0	0	0	0	0	0	0

In Anagrelide-treated patients (*n* = 13), one of each of the following events could be observed: ACS, TIA, Embolic Insult, DVT, PE and MVT. Under Interferon-alpha treatment one PE and one MVT could be observed. Considering Ruxolitinib, also 1 ACS, 1 Embolic Insult, 1 DVT and 1 GI bleeding occurred.

## DISCUSSION

In our observational period of 8 years, 250 patients with suspicious blood cell counts were tested for mutations of JAK-2. 51 (20.4%) patients displayed a JAK-2 V617F mutation (Figure 1). Among the negative patients, 14 were additionally tested for mutations in the CALR gene. Here, no mutations could be found. This relatively small number is due to the fact that CALR mutations have been described to be linked to MPN in 2013 only [9–10] and thus screening was established in our clinical routine only afterwards (from 2014 on).

In our JAK-2 positive patient cohort, we could detect a gender distribution shifting towards men: of the 51 patients, 36 men (70.6%) but only 15 women (29.4%) were diagnosed. Compared with other patient cohorts, this shift is high; for instance a German registry shows more or less equal gender distribution with 51% male MPN patients [41] and also an US-American observational study found only a small overlap of male patients (16332 compared to 15572 female ones) [37].

Divided by subgroups, out of the 51 patients, 33 (64.7%) patients suffered from PV, 15 (29.4%) from ET and 3 (5.9%) from MF (Figure 2). Again here, in all subgroups, male patients were more frequent. For PV, the ratio was 24:9 (2.67:1), for ET 10:5 (2:1) and for MF 2:1 (Figure 3). A male predominance in PV is known from the literature [17], although not to such an extent. For ET, reports state that slightly more women than men are affected [12, 16–17]. Here, our patient cohort displays a complete opposite finding. Concerning MF, literature describes that both genders suffer equally [35]. Again here, our patient cohort showed another distribution pattern; however, our numbers for MF are particularly small. Concluding gender disparities, we could not identify a possible influencing factor yet; however, we plan to further observe this shift, which could diminish with a larger sample.

The median age at diagnosis was 72 years (Table 2, Figure 4), which is in line with other european and international data [12, 16, 37, 41].

Differences in laboratory values were also observed at the time of diagnosis. Disease-typical differences could be observed in the values of RBC (T/l), PLT (G/l) and WBC (G/l; Figure 5): median hematocrit for all MPN patients was 46.25%; 52.5% was the median for PV, 39.9% for ET and 32.0% for MF. Large differences could also be observed in platelet values at diagnosis; 513 G/l was the median value for all patients with MPN: patients suffering from PV were diagnosed with a median value of 453 G/l, patients with ET had 809 G/l and patients diagnosed with MF displayed a median platelet value of 161 G/l. These differences are pathognomonic for the three different clinical groups. Spleen size at the time of diagnosis was not evaluated in our study as this information could unfortunately not be found in the majority of patients’ records at the time of first diagnosis, but was used as disease progression parameter at later time points.

**Figure 6:**
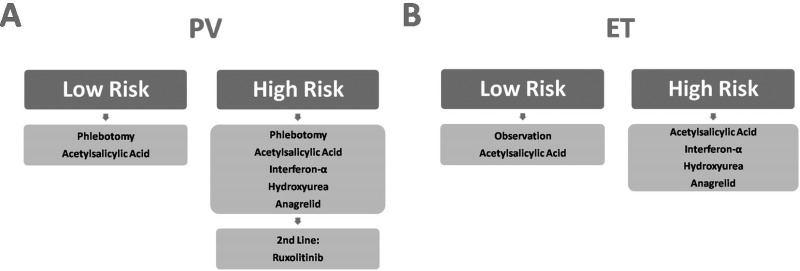
Overview of applied treatment strategies

We also depicted the frequency of different treatments in patients with MPN (Table 4). Due to the long clinical course and disease progression, often multiple treatment options and drugs were applied in one patient. Summarizing, the applied treatment strategy for high risk PV patients was: as first line treatment Phlebotomies and Acetylsalicylic Acid. If Hct values could not be kept under 45%, patients below 70 years of age and without any signs of depression in their medical history received Interferon-α. Alternatively, patients received Hydroxyurea or if Thrombocytosis was the leading symptom Anagrelid. Patients who were intolerant or resistant to Hydroxyurea received Interferon-α during the course of treatment. As second line treatment Ruxolitinib was applied. This treatment strategy can be seen in Figure 6A.

The majority of patients with PV were treated with phlebotomies (72.7%) and/or ASA as a daily therapy (84.8%). Phlebotomies were performed to maintain hematocrit values below 45%. As mentioned above, if this goal could not be reached constantly, patients received Hydroxyurea. This escalation had to be performed in 75.8% of all our PV patients. For patients younger than 70 years or patients who became resistant or intolerant to Hydroxyurea, the therapy of choice in our cohort was Interferon-α, which was the case for 5 patients (15.2%). Furthermore, 6 patients (18.2%) received Anagrelide, a therapy which has been controversially discussed in PV recently, due to possibly increased risk of arterial thrombosis, major bleeding and fibrotic progression [14]. Only 1 patient received therapy with the JAK-2 inhibitor Ruxolitinib. This relatively small number is due to the fact, that Ruxolitinib was approved by the European Medicines Agency (EMA) only in 2013 [42].

None of our patients underwent stem cell transplantation; this is on one hand due to the fact, that at the University hospital Krems no stem cell transplantations are performed, and thus patients are referred to other hospitals. On the other hand, allogeneic stem cell transplantation is regarded in MPN patients only as a therapy option for certain severe cases or after treatment failure [12].

In the disease subgroup of ET, 73.3% of all patients received ASA to reduce the risk of thrombotic events. Furthermore, 66.7% were treated with Hydroxyurea as cytoreductive therapy and 46.7% received Anagrelide to decrease thrombocyte numbers. This treatment algorithm (graphically depicted in Figure 6B) follows again international guidelines, although Anagrelide is controversially discussed in this indication too [14].

In the small subgroup of MF, one patient was prescribed ASA, while the other 2 patients did not receive specific treatment. This was due to high age and multiple co-morbidities, therefore, best supportive care (i.e. blood transfusions, symptomatic therapy) was chosen instead. Here, other registries, like the German SAL-MPN display Hydroxyurea and Ruxolitinib as the most frequently applied drugs in the treatment of MF [41].

In total, 21 vascular events were observed before and 11 after diagnosis in patients with MPN, highlighting the effectivity of our treatment.

In the PV subgroup, the most common vascular complication before diagnosis was ACS with 21.2%. This rate could be reduced to 9.1% during treatment. Interestingly, embolic insults and mesenteric vein thromboses (MVT) occurred only in patients after the diagnosis of PV, however, the relatively small cohort size could be an explanation for this phenomenon. As expected, gastrointestinal bleedings were reported more frequently under therapy, as GI bleedings are unfortunately common side effects of antiplatelet drugs.

One case of each ACS, TIA, PE and hemorrhagic insult (6.7%) was reported in patients with ET prior to diagnosis. In contrast, just one case (6.7%) of PE was observed after the diagnosis.

No vascular events were detected in patients suffering from MF, however one has to bear in mind that this patient subgroup was particularly small (*n* = 3).

We also investigated the correlation between the type of therapy and the specific vascular complications before and after diagnosis of MPN. 25.0% of all patients (*n* = 24) suffering from MPN had an ACS before the diagnosis, while only 12.5% experienced an ACS during the therapy with phlebotomies. This underlines the protective effect of phlebotomies in patients with MPN. There was also a decrease in the number of ACS during the treatment with ASA: 17.9% had an ACS before the diagnosis, whereas only 7.7% suffered from an ACS after diagnosis and initiation of treatment. Phlebotomy and ASA also decreased the number of TIA and other vascular complications in comparison to before diagnosis. However, it is important to note that 20 of the 40 patients treated with T-ASS already received this treatment prior to their MPN diagnosis due to other co-morbidities.

In our study, treatment with Interferon-α (*n* = 7) increased the number of PE, MVT and other vascular events (14.3%) from before to after diagnosis, while the number of TIA, DVT and GI bleeding was reduced under treatment with Interferon-α. 5 people (14.3%) suffered from ACS before the diagnosis, while only 3 patients (8.6%) experienced an ACS under the treatment with Hydroxyurea (*n* = 35). Hydroxyurea could also decrease the number of TIA, hemorrhagic insult and other vascular events, however, it increased the quantity of embolic insult (2.9%) and GI bleeding (5.8%).

1 patient (7.7%) suffered from an ACS and a TIA before the diagnosis and during the treatment with Anagrelide (*n* = 13). Embolic insult, DVT, PE, MVT and GI bleeding were only reported during Anagrelide therapy, but not before the diagnosis, which reflects the controversial debate about Anagrelide [14] in our patient cohort as well.

Stem cell transplantations were not performed at the University hospital Krems, therefore this therapy option could not be well analyzed for vascular complications.

Furthermore, we could not fully assess the length, the strength and the combination effects of different therapies, which were applied to one patient. Also, co-morbidities like diabetes mellitus, arterial hypertension or other risk factors, that favor vascular events, such as smoking, should be considered as biases and could not be displayed in our analysis.

However, a strength of our study is the defined patient cohort and the relatively long observational period.

In conclusion, the major therapy goal for MPN patients is to reduce the risk of vascular events, which could be achieved in the investigated patient cohort. Some further points should be addressed and observed in future studies: the carcinogenicity of Hydroxyurea and Anagrelide should be tested to see if there is propagation in the progression to myelodysplastic syndromes or leukemia and the relatively new drug Ruxolitinib has to be observed and evaluated in long time clinical use.

## MATERIALS AND METHODS

### Patients and clinical data

This study is a retrospective and descriptive analysis of records of patients, who underwent treatment for myeloproliferative syndromes at the Department of Hemato-Oncology of the University hospital Krems, from 2008 to the end of 2015. It aims to determine the number of patients experiencing a vascular event (listed in Table 1) before diagnosis and after diagnosis (during therapy), respectively. Furthermore, the different therapy types were compared with respect to vascular complications in order to evaluate their therapeutic benefits and risks.

The registry was approved by the Ethics Committee of Lower Austria in June 2016 (GS4-EK-4/360-2016), after the content and structure of the study was presented and discussed at an Ethics Committee meeting in St. Pölten.

Inclusion criteria were the following: confirmed diagnosis of MPN with a mutation of either JAK-2 or CALR gene in patients, who have been treated at the University hospital Krems since 2008 and were older than 18 years of age. Exclusion criterion was age under 18.

Screening of eligible patients was performed among all persons that were tested for the respective gene mutations at the University hospital Krems. The clinical data set comprised 250 patients. After diagnosis of MPN, patients were subsequently classified into the 3 disease subgroups (PV, ET and MF). Analyzed data included gender, age, laboratory parameters, medication and vascular events as listed in Table 1. To ensure privacy of the patients, names were blinded with consecutive numbers.

### Molecular analysis

All patient samples were tested for the JAK-2 V617F mutation by Real-Time Polymerase Chain Reaction (RT-PCR; OncoReal JAK2 V617F Real-Time PCR Assay, Ingenetix, Vienna, Austria). From 2014 on, additional testing for CALR mutations was performed; here, Exon 9 of the CALR gene was tested for Deletion 52bp und Insertion 5bp also by means of RT-PCR (OncoReal CALR Type 1 & 2 Assay, Ingenetix, Vienna, Austria).

### Statistical analysis

Clinical data was analyzed with Microsoft Excel, Microsoft Word (Version 15.30) and GraphPad Prism (Version 7.0b).

Parameters analyzed and involved in the study: 1. Demographic Data: a. Age at diagnosis, b. Sex. 2. Disease-specific parameters: a. Haematocrit at diagnosis, b. Number of red blood cells (RBC), white blood cells (WBC) and platelets (PLT) at diagnosis. 3. Specific therapy: a. Type of specific therapy, b. Vascular events before diagnosis (elevated from anamnesis), c. Vascular events after diagnosis/during therapy, d. Vascular events grouped by specific therapy.

## Abbreviations

ACS: Acute coronary syndrome
ASA: Acetylsalicylic acid
CALR: Calreticulin
DVT: Deep vein thrombosis
EMA: European Medicines Agency
EPO: Erythropoietin
ET: Essential thrombocytosis
G-CSF: Granulocyte Colony-Stimulating Factor
G/l: Giga per liter (10^9^ cells per liter)
GI: Gastrointestinal
Hct: Hematocrit
JAK-2: Januskinase-2
MF: Myelofibrosis
MPL: Myeloproliferative Leukemia gene
MPN: Myeloproliferative neoplasm
MVT: Mesenteric vein thrombosis
PCR: Polymerase Chain Reaction
PE: Pulmonary embolism
PLT: Platelets
preMF: Prefibrotic Myelofibrosis
PV: Polycythemia vera
RBC: Red blood cells
RT-PCR: Real-Time Polymerase Chain Reaction
T/l: Tera per liter (10^12^ cells per liter)
TIA: Transitory ischemic attack
TPO: Thrombopoietin
WBC: White blood cells
WHO: World Health Organization
